# Dissecting early life stress-induced adolescent depression through epigenomic approach

**DOI:** 10.1038/s41380-022-01907-x

**Published:** 2022-12-14

**Authors:** Shinichiro Ochi, Yogesh Dwivedi

**Affiliations:** 1 Department of Psychiatry and Behavioral Neurobiology, University of Alabama at Birmingham, Birmingham, AL 35294 USA; 2 Department of Neuropsychiatry, Molecules and Function, Ehime University Graduate School of Medicine, Shitsukawa, Toon, Ehime 791-0295 Japan

**Keywords:** Depression, Neuroscience

## Abstract

Early life stress (ELS), such as abuse and neglect during childhood, can lead to psychiatric disorders in later life. Previous studies have suggested that ELS can cause profound changes in gene expression through epigenetic mechanisms, which can lead to psychiatric disorders in adulthood; however, studies on epigenetic modifications associated with ELS and psychiatric disorders in adolescents are limited. Moreover, how these epigenetic modifications can lead to psychiatric disorders in adolescents is not fully understood. Commonly, DNA methylation, histone modification, and the regulation of noncoding RNAs have been attributed to the reprogramming of epigenetic profiling associated with ELS. Although only a few studies have attempted to examine epigenetic modifications in adolescents with ELS, existing evidence suggests that there are commonalities and differences in epigenetic profiling between adolescents and adults. In addition, epigenetic modifications are sex-dependent and are influenced by the type of ELS. In this review, we have critically evaluated the current evidence on epigenetic modifications in adolescents with ELS, particularly DNA methylation and the expression of microRNAs in both preclinical models and humans. We have also clarified the impact of ELS on psychiatric disorders in adolescents to predict the development of neuropsychiatric disorders and to prevent and recover these disorders through personalized medicine.

## Introduction

Early life is a highly sensitive and critical period for brain development, cognitive maturation, and the formation of synaptic structures and synaptic interconnections in the central nervous system (CNS) [1]. Adverse experiences in this period may profoundly impact these processes and can lead to psychiatric disorders in adult life. In the USA, more than 180,000 child abuse cases were reported in 2017 [2]. Early life stress (ELS), such as physical abuse, sexual abuse, emotional abuse, physical neglect, and emotional neglect, are known as significant risk factors for many adverse health problems during adulthood [3–5] and are associated with physical illnesses such as diabetes, cardiovascular disease, and malignant tumor [6–8]. Exposure to ELS is a significant risk factor for mental illnesses such as major depressive disorder (MDD), schizophrenia, bipolar disorder, borderline personality disorder, posttraumatic stress disorder (PTSD), and substance use [9–24]. The number of early life adverse experiences is also correlated with the increased risk of depression during adulthood. For example, there is a 4-times higher risk of depression in a person who had multiple episodes of early-life adversity than in someone who had not experienced any early-life adversity [25].

In childhood and adolescence, ELS is not only associated with the heightened risk of adverse health problems [26–32], including physical illnesses such as headache [33] and asthma [34] but also mental illnesses such as depression, non-suicidal self-injury (NSSI) [35–49]. A meta-analysis that examined the association between ELS and depression in adolescents found that the association between childhood abuse/neglect and depression was much stronger in adolescents than in adults [50]. More recently, another meta-analysis performed by LeMoult et al. [51] estimated the associations between ELS and the risk for the onset of MDD before the age of 18 years. These authors also examined the associations between a specific type of ELS and risk for youth-onset MDD. They found that individuals who experienced ELS were more likely to develop MDD before the age of 18 years than individuals without a history of ELS. Whereas some types of ELS were not associated with MDD, other types (emotional abuse) were more strongly associated with MDD. It is also suggested that the impact of ELS on adolescents is sex-dependent. For example, a study reported that in young adulthood, the mental health of females with ELS was worse than males with ELS, and males with ELS were associated with more substance use than females with ELS [52].

Suicidal behavior, such as suicide attempts and suicide ideation, is one of the most important psychiatric problems. Affective temperamental dysregulation might be a possible contributor to adverse clinical outcomes in depressed patients [53]. In this context, it is important to note that ELS is significantly associated with suicide attempts [54, 55], suicide ideation [56, 57], and a high risk of premature mortality, including completed suicide [58] later in life. Among various early life stressors, sexual abuse has been strongly associated with suicidal behavior [59, 60] during adulthood. In addition, childhood physical abuse and witnessing domestic violence are significantly linked with a higher risk for both suicidal ideation and attempts [61] in adults. Other studies have also shown an association between ELS and suicide attempts with various psychiatric disorders [62–65]. Interestingly, patients with mental illnesses who had a history of ELS are a biologically and clinically different subtype with greater symptom severity, poorer treatment outcome, and greater risk for suicide compared to patients who did not have a history of ELS [66]. A recent meta-analysis reported that emotional abuse, physical abuse, and sexual abuse were significantly associated with an elevated risk for suicide attempts in adults [67]. These studies suggest that a specific type of ELS could be specifically associated with suicidal behavior in the adult population.

As with the adult population, ELS is also linked with suicidal behavior in adolescents [68]. Interestingly, females with ELS were more significantly associated with suicide attempts than males with ELS in their early teens [69]. Stratifying the sex differences, one study reported that physical, emotional, and sexual abuse, household dysfunction, psychological symptoms, and lower social support were significantly correlated with suicide attempts in both boys and girls [69]. However, being younger age, and having physical and emotional neglect experiences increased the risk of suicide attempts in girls only. When exposed to physical abuse, being younger age and having lower social support, girls were more likely to have suicide attempts than boys. Conversely, suicide attempts were more among boys than girls when exposed to psychological symptoms. This suggests that the effect of ELS could be sex-specific. Whether this association is specific to the younger population or common to adults, needs to be further studied.

Given the prevalence and substantial association of ELS with depression and suicidal behavior in adolescence, there is an urgent need to identify neurobiological factors that can contribute to these disorders in youth. In this review, we have critically evaluated the current knowledge of genetic and epigenetic factors that can contribute to the development of depression in the adolescent/youth population. Emphasis has been given to epigenetic factors, given that they are significantly affected by the environment. In addition, we have also discussed the possibility of epigenetic modifications being used as effective targets for diagnostic biomarkers or therapy.

### Genetic contributors of ELS-induced depression in adolescents

One of the most common studies in ELS is the study of single-nucleotide polymorphisms (SNPs). Many previous studies have reported an association between brain-derived neurotrophic factor (BDNF) and psychiatric disorders such as depression and suicide in the adult population [70–72]. However, no significant differences were found in BDNF levels in adolescents with sexual abuse with or without PTSD [73]. Interestingly, in patients with PTSD, decreased cortisol levels were found with increasing time after trauma, and no significant correlation was found with the cortisol levels in patients without PTSD, suggesting that cortisol may play a role in individuals who sustained sexual assault. In the BDNF gene, the SNP, which is the substitution from valine (Val) to methionine (Met) in the functional coding region at codon 66 (BDNF Val66Met), has received the most attention in mental disorders, including depression [74]. Several studies have shown an association between BDNF Val66Met polymorphism and stress responses of ELS [75–84] and PTSD [85] in adolescents. A study by Chen et al. [84] in 780 pairs of ethnic Han Chinese adolescent twins showed that the frequency of stressful life events was significantly correlated with depressive symptoms, but there was no significant main effect on the *BDNF* Val66Met genotype. On the other hand, the interaction between the *BDNF* Val66Met genotype and stressful life event frequency was significant. Individuals with one or two Val alleles demonstrated a greater susceptibility to both the detrimental effects of higher stress and the beneficial effects of lower stress compared to the Met/Met genotype. Another study followed 889 mothers and their children from 3 months to 12 years and examined an association between BDNF Val66Met and behavior problems in adolescents [86]. Information on maternal depressive symptoms was gathered postpartum and at a 12-year follow-up. The results showed a significant association between maternal symptoms of depression and anxiety and the tendency to internalize problems in 12-year-old children. Surprisingly, maternal depressive and anxious symptoms, such as pre-maternal stress, were not associated with BDNF Val66Met and behavior problems in adolescents.

The SNPs of the promoter region of the serotonin transporter gene (5HTTLPR) have not only been studied in adolescent depression with ELS [76, 81, 86] but also with suicidal behavior [87]. In adolescent depression, a significant main effect and a gene-environment interaction effect of the short (SS) allele were found only among females [88], suggesting sex differences in interaction effects between the 5HTTLPR polymorphism and maltreatment in the prediction of adolescent depression. The role of stress, social support, and the short allele of the 5-HTTLPR and the met allele of the BDNF have also been studied in children following a natural disaster [85]. BDNF analyses showed several gene × environment interactions. Greater stress was related to more symptoms of PTSD and depression, and this effect was stronger in children with the met allele. No changes were found for 5-HTTLPR. Some studies have also reported other genes, such as the mineralocorticoid receptor gene (NR3C2) [89], the promotor of serotonin transporter gene (SLC6A4) [82], and FK506-binding protein 5 (FKBP5) [90, 91] with ELS and depression.

As discussed above, a majority of the studies have taken a candidate gene approach. Most of these have focussed on *BDNF* gene and, to a certain extent, stress-related genes. To the best of our knowledge, there is no meta-analysis that has analyzed the associations between ELS, epigenetics, and adolescent depression or suicide. Candidate gene studies generally focus on known genes that are thought to be involved in the pathogenesis of the disease and compare the frequency of the gene polymorphisms between patients with the disease and controls. These are generally hypothesis-driven, and the advantage is that it allows to compare the findings accumulated by various studies to consider the direction of the research and to accumulate more data steadily. However, biases can easily arise with candidate gene studies. On the other hand, genome-wide association studies (GWAS) cover almost the whole genome and statistically examine the association between SNP frequencies and the disease. These studies are data-driven without a hypothesis in advance, analyze the results, and then proceed with the study to identify SNPs or other variants in the DNA that are associated with the disease. So far, there is no GWAS examining the association between ELS and adolescent depression. Future studies should be directed in this context. In addition, recent molecular genetic studies indicate that the genetic basis for complex traits such as MDD is polygenic, which results from a combination of many genetic variants [92]. Usig a polygenic approach, a recent study examined the interaction between genetic risk for MDD and a multi-informant longitudinal index of critical parenting in relation to depressive symptom development from early to late adolescence [93]. The Latent Growth Model suggested that polygenic risk score for MDD was associated with higher depressive symptoms across adolescence, particularly for those experiencing elevated levels of critical parenting. The authors highlighted how polygenic risk, in combination with a general environmental factor, can influence depressive symptom development from early to late adolescence. Similar approaches need to be used with respect to ELS and depression in both adults and adolescents and examine how they are commonly associated or differ from each other in the development of MDD in these two populations.

### Epigenetics and their association with ELS and depression in adolescents

**Fig. 1:**
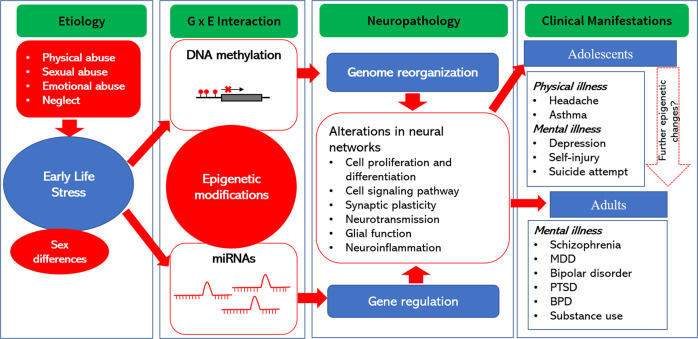
Early life stress mediated epigenetic modifications as regulators of neuropsychiatric disorders in adolescence. Early life stress such as physical abuse, sexual abuse, emotional abuse, and neglect leads to epigenetic modifications such as DNA methylation and abnormal microRNA expression, which causes genome reorganization and alterations in gene regulation in a sex-dependent manner. These changes may affect neural networks leading to aberrant cell proliferation, synaptic plasticity, neurotransmission, and neuroinflammation. This may lead to physical and mental illnesses in adolescents and adulthood. Clinical manifestations in adolescents can cause further epigenetic modifications and affect neuropsychiatric disorders in adulthood. G × E gene and environmental, MDD major depressive disorder, miRNA microRNA, PTSD posttraumatic stress disorder, BPD borderline personality disorder.

Epigenetics in the context of ELS and psychiatric illnesses during adolescence is an emerging field of research. Epigenetics refers to long-standing gene expression changes regulated via transcriptional, post-transcriptional, translational, and/or post-translational mechanisms such as DNA methylation, DNA hydroxymethylation, and histone modifications, which do not entail any change in DNA sequence. These epigenetic changes have been widely reported in various psychiatric conditions, including suicidal behavior [71, 94–96], as well as a biomarker in treatment response [97]. Several recent reviews have highlighted the importance of epigenetics in ELS-induced behavioral changes in humans and animals [6, 98–104]. A schematic representation of the impact of ELS on genome organization and gene regulation is depicted in Fig. 1. In this review, we aimed to evaluate the current evidence on epigenetic modifications in adolescents with ELS and to explore the possibility of them being used as effective targets for diagnostic biomarkers or therapy.

### DNA methylation

DNA methylation is one of the most studied epigenetic mechanisms regarding psychiatric illnesses. DNA methylation, defined as the addition of the methyl group on the fifth carbon of cytosines (5-methyl-cytosine (5mC)), commonly occurs in life, including the development process. DNA methylation is reported at CpG sites at gene promoters and is associated with transcription silencing in mammalian genomes [101]. The functions of 5mC methylation in different regions of the genome, except for the promoter regions, are not fully understood [101]. Early studies focused on CpG islands (CGI) representing DNA regions of a high CpG density, which were shown to be low or unmethylated. Recent work, however, has shown that DNA methylation can also directly silence genes with non-CGI promoters. In DNA methylation at promoter regions, three processes are involved: de novo DNA methylation, maintenance, and demethylation [105]. DNA methyltransferase enzymes transfer the methyl group from S-adenosyl-L-methionine to cytosine, whereas Ten-eleven-translocation proteins methylcytosine dioxygenases and thymine DNA glycosylase execute active demethylation. In the demethylated gene body, CG-rich promoter, which is highly methylated, is silenced [99, 104, 105].

A few studies suggest that DNA methylation plays a crucial role in adolescent depression. For example, in 25 adolescents and 20 healthy controls, Chiarella et al. [106] examined resting-state assessments and brain morphometry along with salivary *SCL6A4* and *FKBP* methylation. They found that *SCL6A4* methylation was linked to amygdala-frontal operculum resting-state functional connectivity, regardless of diagnosis, and was differentially associated with inferior orbitofrontal gyrus gray matter volume in adolescents with depression and control subjects. On the other hand, *FKBP5* methylation was associated with inferior orbitofrontal gyrus gray matter volume in depressed and healthy adolescents and orbitofrontal cortex rostral prefrontal cortex connectivity only in healthy adolescents. These data show that *FKBP5* and *SLC6A4* methylation levels are associated with brain connectivity and structure in regions relevant to adolescent depression. Raffetti et al. [107] reported glucocorticoid receptor (*NR3C1*) DNA methylation was significantly associated with a higher risk for substance use in adolescents, and *NR3C1* exon 1F locus hypermethylation can predict substance use in adolescents. Roberson-Nay et al. [108] investigated DNA methylation from 150 Caucasian monozygotic adolescent twins with or without major depression. About 760 differentially and variably methylated probes/regions mapped to 428 genes with early-onset depression were detected. Genes previously implicated in mood and psychiatric disorders, as well as chronic stress (e.g., *NRG3*) were also identified. Gene enrichment analyses implicated genes related to neuron structures and neurodevelopmental processes, as well as cell adhesion.

Although limited in number, DNA methylation studies are emerging and seem to be critical in adolescent depression. How they are different from adult depression, remains to be seen. Future follow-up studies with repeated measures from childhood to adulthood in individuals with a depressive disorder are needed to understand how the relationship between DNA methylation and brain processes changes throughout the lifespan.

#### Studies of DNA methylation in adolescents with ELS

##### Animal studies

Animals have been extensively used to investigate the effects of ELS on epigenetic modifications. Maternal separation (MS) is one of the most popular ELS models [109–113]. In this model, neonatal pups are separated from their mothers for a certain period of time every day for the first 2 to 3 weeks [6]. This results in inconsistent maternal care provided to the pups, which ultimately leads to long-term anxiety-like and depressive-like behaviors. Jaric et al. [114] used C57BL/6 mice to establish a two-hit developmental stress model, including MS in early life followed by social isolation in adolescence. They found that single exposure to early-life stress had the most significant impact and was female-specific in generating anxiety- and depression-related phenotypes. They also reported that transcriptional and methylation alterations in psychiatric risk genes, *Nr3c1*, and calcium voltage-gated channel subunit alpha1C (*Cacna1c*), most likely contributed to the stress-induced behavioral effects in these animals. Leussis et al. [115] used the triadic model of learned helplessness to understand controllability, helplessness, and motivational factors following maternal separation in male and female adolescent rats. They found sex-dependent changes, with males demonstrating loss of controllability in an escapable shock condition, whereas females demonstrating motivational impairment in a no-shock condition. Although no epigenetic studies were done in these animals, the authors noted reductions in parvalbumin, a GABAergic marker, in the prefrontal cortex of separated rats relative to age-matched controls and paralleled depressive-like behavior. This model seems promising and can be used to further study epigenetic consequences during adolescence.

Chronic mild stress has also been used as an ELS model [116, 117]. Deng et al. [118] investigated Sprague Dawley rats who received predictable chronic mild stress in adolescence. The study revealed that DNA methylation in exons IV and VI of BDNF were significantly decreased compared to the control. Yang et al. [119] investigated FKBP5 DNA methylation in the hippocampus from C57BL/6J mice, that were administered corticosterone from 5 weeks old for four weeks, as a stress model and revealed intron5 of FKBP5 DNA methylation was significantly decreased compared to controls. In male mice, maternally separated for four hours a day from P10 to P17 combined with limited nesting material, Kronman et al. [120] recently reported downregulation of H3K79me2 in dopamine D2 median spiny neurons of the nucleus accumbens along with altered expression of DOT1 like histone lysine methyltransferase (Dot1L) and lysine demethylase 2b (Kdm2B), enzymes that control this modification.

Recently, Fitzgerald et al. [121] used a modified maternal separation (MMS) model, which involved repeated stimulation of pups for 1.5 h/day, while separated from their mother from postnatal day (P) 4-6. 3′mRNA and DNA methylation immunoprecipitation sequencing were performed on hypothalamic tissue at P6. The authors found that although MMS was associated with subtle changes in gene expression, there were widespread alterations in DNA methylation along with hyperactivity in the elevated plus and open field mazes. They concluded that ELS had marked effects on DNA methylation in the hypothalamus in early life, resulting in stress-specific hyperactivity in young adulthood. In another study, Seo et al. [122] examined the effects of ELS on hippocampal S100 calcium-binding protein A10 (p11) expression, histone acetylation, and DNA methylation at the p11 promoter at different stages of adulthood. Pups were subjected to MS for 3 h daily from postnatal day 1 to 21. At young and middle adulthood, behavioral tests were measured. Mice in both age groups showed reduced hippocampal p11 levels, a decrease in histone acetylation (AcH3), and permissive histone methylation (H3K4me3) at the p11 promoter, as well as an increase in repressive histone methylation (H3K27me3). In addition, AcH3 and H3Kme3 levels of the p11 gene in response to MS were reduced with age. DNA methylation analysis of the p11 promoter revealed increased CpG methylation in middle-aged MS mice only. The results show the age-dependent negative effects of ELS on the epigenetic modifications of p11 transcription.

Altogether, the animal studies demonstrate that regardless of the animal models used, ELS can have widespread epigenetic effects during the adolescent phase; some of them are correlated with behavioral responses. Since animal studies have used rats of different strains as well as different models of ELS, more replication studies are needed. Furthermore, epigenetic modifications vary with brain regions, and some of the changes are cell type-specific; further studies will be required to examine these epigenetic changes in depth.

##### Clinical studies

Several clinical studies have examined an association between DNA methylation and ELS in the adolescent population. Cecil et al. [123] collected buccal epithelial cell DNA from 124 adolescents and youth—68% of whom reported experiencing some form of maltreatment during early life and investigated the array-based genome-wide methylation. The strongest association between methylomic variations and ELS was noted for physical abuse. Many identified loci were annotated to genes previously implicated in stress-related outcomes. For example, there was a significant association between physical abuse and presenilin 2 (PSEN2), sexual abuse and glutamate ionotropic receptor NMDA type subunit 2D (GRIN2D), and physical neglect and synaptojanin 2 (SYNJ2). Gene ontology analyses revealed that different types of ELS not only showed unique methylation patterns enriched for specific biological processes but also shared a common epigenetic signature, primarily related to neural development and organismal growth. The data suggest that epigenetic changes can distinguish the type of ELS and psychopathology.

Since epigenetic modifications are tissue-specific, Nieratschker et al. [124] applied a unique cross-species and cross-tissue approach to examine ELS-induced epigenetic changes. They reported that several regions in *MORC1* (MORC family CW-type zinc finger 1) were differentially methylated in response to ELS in CD34+ cells and CD+ T cells derived from the blood of human and monkey neonates, as well as in CD3+ T cells derived from the blood of adolescent monkeys and in the prefrontal cortex of adult rats. A gene-set analysis from a genome-wide association study also suggested an association of *MORC1* with MDD. This study is the first to identify epigenetic marks on a gene present in blood cell progenitors at birth and in the brain in adulthood, which shows an association with depression.

In cord blood from newborns with ELS in the prenatal stage, Devlin et al. [125] found significantly decreased promoter DNA methylation of the serotonin transporter gene (SLC6A4). NR3C1 DNA methylation from cord blood was also associated with newborns with ELS [126, 127]. Unternaehrer et al. [128] examined whether maternal adversities and cortisol levels during pregnancy predict cord blood DNA methylation of the oxytocin receptor (*OXTR*) and found that *OXTR* methylation was significantly associated with newborns with ELS in the prenatal stage. Interestingly, the number of stressful life events and maternal cortisol profile were negatively associated with *OXTR* DNA methylation. This suggests that distinct prenatal adversities predict decreased DNA methylation in the *OXTR* gene, which is relevant for childbirth, maternal behavior, and the well-being of mother and offspring.

Since childhood trauma affects social cognition and the basic processing of social cues, studies have examined whether epigenetic changes in the *OXT* gene contribute to long-term behavioral effects. Lesemann et al. [129] examined the N170 response to neutral faces in relation to participants’ recalled childhood trauma and methylation of oxytocin structural (*OXTg*) and oxytocin receptor (*OXTRg*) genes. They reported that *OXTg* and *OXTRg* methylation in female adolescents with ELS were associated with electroencephalographic N170 response to faces, which is a measure to capture neural response, and predicated a weakened N170 response in those with high methylation, and hyper-vigilance with participants with low methylation. Nishitani et al. [130] reported that DNA methylation-based age in adolescents with ELS was significantly increased compared with healthy controls using Pediatric-Buccal-Epigenetic (PedBE) clock methods, suggesting that PedBE age acceleration can be widely used as a biological marker for predicting atypical developmental features, including developmental vulnerabilities caused by maltreatment.

Sumner et al. [131] collected 113 saliva samples from adolescents (ages 8–16 years) with ELS reflecting the dimensions of threat and deprivation and applied the Illumina EPIC BeadChip array. Adjusting for lifetime experience of neglect, lifetime experience of abuse was associated with DNA methylation at 4 CpG sites (cg20241299, cg08671764, cg27152686, and cg24241897), whereas, adjusting for abuse, DNA methylation was associated with one CpG site (cg03135983). This study provides crucial evidence that DNA methylation changes over time with ongoing adverse experiences and that these experiences are characterized by distinct DNA methylation patterns. Grasso et al. [132] reported that FKBP5 DNA methylation in newborns with the T allele in SNPs was associated with maternal threat-related ELS, such as violence, but not with deprivation-related ELS, such as neglect. In an interesting study, Serpeloni et al. [133] examined the molecular mechanisms that mediate long-term consequences of early stress across generations. They determined the genome-wide DNA methylation profile in 121 children and tested for associations with exposure to grandmaternal interpersonal violence during pregnancy. They found methylation variations of five CpG sites significantly associated with the grandmother’s report of exposure to violence while pregnant with the mothers of the children, supporting the idea that DNA methylation may serve as a biological mechanism in the transmission of stress across generations.

Although many previous clinical studies have reported an association between DNA methylation and mental disorders in adult patients with ELS, to the best of our knowledge, there are only a few studies pertaining to DNA methylation and mental disorders in adolescents with ELS. Weder et al. [134] examined whether epigenetic markers can predict dimensional ratings of depression in maltreated children. Using a genome-wide methylation study, they found that the changes in DNA methylation of DNA-binding protein inhibitor ID-3 (ID3), glutamate receptor, ionotropic N-methyl-D-aspartate (NMDA) 1 (GRIN1), and tubulin polymerization promoting protein (TPPP) were predictors of depression in these children. DNA methylation in FKBP5 was also found to be associated with the rs1360780 SNP in bipolar disorder with ELS [135]. Yang et al. [136] collected saliva DNA samples from 96 adolescents with ELS and 96 controls and investigated the array-based genome-wide methylation. They revealed significant differences in DNA methylation at 2868 CpG sites between adolescents with ELS and controls. In blood samples from 46 adolescents with ELS, Radtke et al. [137] found that glucocorticoid receptor (hGR) gene methylation was associated with mental disorders in adolescents with ELS, especially cg1760381 methylation was associated with symptoms of borderline personality disorder. In an interesting study, Kaufman et al. [138] examined an association between DNA methylation and fMRI outcomes in 157 adolescent subjects with ELS. They reported that orthodenticle homeobox 2 (*OTX2*) methylation was associated with the right ventral medial prefrontal cortex and bilateral regions of the medial frontal cortex and cingulate cortex. Efstathopoulos et al. [139] collected saliva DNA from 1149 adolescents and revealed that exon 1 of NR3C1 methylation was significantly associated between depressive symptoms and ELS. In saliva samples from 247 adolescents with ELS, Sumner et al. [140] investigated methylation age using array-based genome-wide methylation. The threat-related ELS was uniquely associated with older DNA methylation age, but not deprivation-related ELS. Interestingly, depressive symptoms were associated with older DNA methylation age. This study suggests that early threat-related experiences may particularly be associated with accelerated biological aging in youths, which may be a mechanism linking ELA with depressive symptoms.

Serpeloni et al. [141] studied psychiatric illnesses and genome-wide DNA methylation following intimate partner violence (IPV) during pregnancy. They found that mothers and children with IPV had elevated depression, PTSD, and anxiety symptoms. Surprisingly, when IPV occurred during and after pregnancy, these problems were absent in children. They revealed that following prenatal IPV, DNA methylation in NR3C1 and FKBP5 genes were most methylated in adolescents with ELS. These children also showed more DNA methylation in heterochromatin-like regions, previously associated with stress/disease resilience. These results indicate an enhanced ability to terminate hormonal stress responses in prenatally stressed children and provide novel insights on how prenatal stress may epigenetically shape resilience in humans.

The studies mentioned above provide a glimpse into the use of various epigenetic approaches in examining ELS and associated psychiatric illnesses. Given the use of varied subject populations and methods to evaluate behavioral responses, it is challenging to assess uniformity in the reported findings; nevertheless, these studies clearly show that DNA methylation plays a crucial role in ELS-induced behavioral changes. A large number of studies have focused on *FKBP5* and *NR3C1* and *OXT* gene, broadly associated with stress social and emotional behavior, respectively, and found a consistent response to ELS and depression. Similar findings have been reported in the adult population [142]. Whether these effects are broadly associated with ELS and depression, regardless of age, needs further study.

##### MicroRNAs as mediators of ELS and depression

MicroRNAs (miRNAs) are a class of single-stranded small non-coding RNAs that act as an important epigenetic modifier and regulate protein levels of target messenger RNAs (mRNAs). They play vital roles in a variety of developmental processes, including cell proliferation, differentiation, synaptogenesis, synaptic plasticity, and apoptosis [143, 144]. MiRNAs are generated from short hairpin RNAs by two ribonuclease III-type proteins and, as the name indicates, are short in length (~22 nucleotides) [145]. By altering the intracellular stability of mRNAs post-transcriptionally, miRNAs regulate protein-coding genes and, subsequently, protein production [6, 97]. MiRNAs are synthesized in the nucleus where primary miRNA (pri-miRNA) with a hairpin loop structure is produced from the transcription of the miRNA gene by RNA polymerase II. Pri-miRNA is cleaved into precursor miRNA (pre-miRNA) by Drosha ribonuclease III (DROSHA) and Di George syndrome critical region in gene 8 (DGCR8). Pre-miRNA is then transported from the nucleus to the cytoplasm by Exportin 5 (EXPO-5), where pre-miRNA is converted into miRNA duplex by Dicer, an endonuclease cytoplasmic RNase III enzyme, and trans activation response RNA-binding protein (TRBP). MiRNA duplex is loaded onto an Argonaute (AGO) protein where only one strand is selected by the AGO protein as the mature miRNA. The mature miRNA with AGO protein forms the RNA-induced silencing complex (RISC). Post-transcriptional modifications of coding genes mediated by miRNAs generally happen through RISC, which induces target mRNA degradation or translational repression [145]. MiRNA binding sites are located in the 3′ untranslated region (UTR) of target mRNAs which is determined by their 5′ end from 2 to 7 nucleotide position as miRNA seed [145, 146].

In the past few years, a considerable amount of research has been conducted to examine the role of miRNAs in neuropsychiatric disease [147–156]. We and other investigators have examined the expression of miRNAs in human postmortem brains of depressed subjects, in the brain of animals showing depression-like behavior, and in peripheral tissues such as blood, urine, and saliva [6, 96, 144, 150, 157–167]. We have demonstrated that miRNAs form highly correlated networks in the brains of MDD subjects that differ from healthy controls, suggesting that miRNA networks can give rise to specific behavioral phenotypes [96, 168, 169]. We have also shown that rats who display hopelessness (i.e., learned helplessness), a clinical phenotype of suicide risk, has a blunted frontal cortical miRNA response to acute stress compared to non-hopeless rats [170], suggesting that aberrant miRNA expression can lead to deficits in the coping response to stress. Genetic differences in miRNA expression can also influence the coping response to a stressor. The stress-sensitive F344 rats that have an exaggerated release of corticosterone (CORT) to stressors show increased expression of hypothalamic miR-18a that binds to 3’UTR of glucocorticoid receptor and reduces its expression [171]. This results in increased CORT release by reduced feedback regulation. In addition, exposure of neurons to excessive CORT results in a decrease in the BDNF-dependent expression of postsynaptic proteins via suppression of miR-132 [172]. MiRNAs dynamically fluctuate, temporally and spatially, throughout the lifespan of an organism. For example, two studies focusing on Lin28/let-7 system in rodents and monkeys found that the expression of Lin28a and Lin28b from neonatal/juvenile to adult ages were significantly different compared with adults [173, 174]. Let-7b had the largest increase between puberty and adulthood in rats, increasing by 400%. Significant expression changes in miR-9, miR-132, and miR-145 were also observed between neonatal to adult ages.

MiRNAs are also released into the blood and CSF [175–177]. Circulatory miRNAs are stably expressed under healthy conditions, but the miRNA profile changes dramatically under pathological states, suggesting that peripheral miRNAs can be used as disease biomarkers [178, 179], including psychiatric illnesses [164, 180–183].

#### ELS Studies of miRNAs

##### Animal studies

A detailed review of literature has recently been published by us, highlighting the role of miRNAs in ELS [6]. More recently, our group used MS as a rodent model of ELS and tested whether miRNAs target serotonin genes to regulate ELS-induced depression-like behavior and whether this effect is sex-dependent. We also examined whether environmental enrichment prevents susceptibility to depression- and anxiety-like behavior following MS and whether enrichment effects are mediated through serotonin genes and their corresponding miRNAs [184]. It was observed that MS decreased sucrose preference, which was reversed by enrichment. Males also exhibited greater changes in forced swim climbing and escape latency tests only following enrichment. Expression levels of serotonin transporter *Slc6a4* and 5-hydroxytryptamine receptor 1A (*Htr1a*) were upregulated in the frontal cortex following MS. In male MS rats, enrichment reversed *Htr1a* expression to levels similar to control rats. MiR-200a-3p and miR-322-5p, which target *SLC6A4*, were decreased by MS. An *HTR1A*-targeting miRNA, miR-320-5p, was also downregulated by MS and showed slight reversal by enrichment in male animals. MiR-320-5p targeting of *Htr1a* was validated in vitro using SHSY neuroblastoma cell lines. Altogether, this study implicates miRNA interaction with the serotonin pathway in ELS-induced susceptibility to depression-related reward deficits. Furthermore, because of its recovery by enrichment in males, miR-320 may represent a viable sex-specific target for reward-related deficits in major depressive disorder.

Our group also tested the expression of miRNAs in the hypothalamus following ELS and susceptibility to depression-like behavior and whether sex or acute stress exacerbates this response [185]. We further tested whether environmental enrichment promotes early life and adult behavioral stress resilience and its effect on hypothalamic miRNA and gene expression. Interaction of MS, enrichment, restraint stress, and sex showed alterations in miRs-29, -124, -132, -144, and -504. Sex had a significant effect on a large number of miRNAs. Also, environment enrichment reversed the downregulation of miR-29b-1-5p and -301b-3p in MS. qPCR analysis showed that MAPK6 and MMP19, targets of miR-301b-3p, were upregulated in MS and reversed by enrichment. Additionally, miR-219a was hypermethylated in MS, coinciding with decreased miR-219a expression. This study suggests that sex plays a critical role in the hypothalamic miRNA response to both ELS and acute stress, with males expressing greater changes following postnatal stress. Moreover, enrichment significantly altered behavior as well as hypothalamic miRNA expression and their gene targets.

Using unpredictable chronic mild stress in adolescence as an ELS model, Guo et al. [186] investigated the effects of miR-15b using C57 GAD67-GFP mice. They found that the injection of miR-15b antagomir significantly improved depression-like behavior and improved the reductions of excitatory synapse and syntaxin-binding protein 3 (STXBP3A)/vesicle-associated protein 1 (VAMP1) expressions in ELS model. The injection of a miR-15b analog into the nucleus accumbens induced similar behavior and down-regulation of STXBP3A/VAMP1 expressions in ELS mice.

##### Clinical studies

Clinical studies of miRNAs in ELS are quite limited. In patients with bipolar disorder and history of ELS, Prados et al. [187] reported that hyper-methylation miR-124 promoter was correlated with ELS history and symptom severity compared to depressed patients with no ELS history. In a sample of 32 controls (11 with and 22 without an early trauma history), Cattane et al. [188] reported differential regulation in 80 miRNAs in the ELS group compared with participants without ELS. MiR-29b-3p, miR-29c-3p, and miR-16-5p were significantly upregulated, while miR-200b-5p and miR-125b-1-3p were significantly downregulated. In prenatally stressed rodents, these authors found a decrease only in miR-125-1-3p, suggesting that miR-125-1-3p may be specifically responsive to ELS, and the effects are lasting and consistent across species. In another study, Suderman et al. [189] reported analyzed the methylation of 489 miRNAs using methylated DNA immunoprecipitation and revealed that an altered pattern of methylation in 39 miRNAs was associated with ELS. Confirmatory analysis showed that miR-514, let-7d, miR-520c, miR-215, miR-519a, and miR-519e were hypermethylated, whereas miR-203 was hypomethylated. So far, there are only a few clinical studies examining the relationship between miRNA and mental disorders in adolescents with ELS. Ran et al. [161] collected miRNAs from serum extracellular vesicles and did genome-wide miRNA sequencing in a sample of 17 adolescents (9 untreated MDD subjects and 8 controls) and validated it in a sample of 72 adolescents (34 untreated MDD subjects and 38 controls). They identified 18 upregulated miRNA and 14 downregulated miRNAs in MDD subjects when compared with controls and revealed that the expressions of miR-450a-2-3p, miR-556-3p, and miR-2115-3 were significantly different, and there was an association between miR-450a-2-3p and ELS.

MiRNAs in the field of adolescent depression are an emerging area of research. As described above, both animal and human studies indicate that several miRNAs are responsive to ELS. Whether these miRNAs can serve as vulnerability factors in the development of depression associated with ELS is not clearly known; however, as shown by us [185], the expression of miRNAs is significantly altered in adult MS rats who showed depression phenotype, and these changes are sex-specific. Further studies will be required to examine the impact of miRNAs on ELS and their susceptibility to developing depression during adolescence. Also, comparing adult and adolescent subjects with depression who had prior experience with trauma will determine if miRNA changes are similar across age groups.

## Conclusions and future directions

A summary of findings pertaining to ELS and its impact on DNA methylation and miRNAs is provided in Tables 1 and 2. As can be seen, whereas there have been several studies on the effect of ELS on epigenetic modifications in adulthood, only a handful of studies are available showing such effects in adolescents, especially in human subjects. Also, there are only a few studies that have examined associations between the frequency or intensity of ELS, epigenetic changes, and subsequent onset of adolescent depression. In addition, there is no large-scale study in depressed patients who had gone through other stresses from the time of the ELS to the time when they were studied. Thus, it is difficult to dissect an association between the initial effects of ELS on epigenetic changes and those that occur over a period of time during adolescent depression. Further clinical longitudinal studies of the impact of each patient’s life events, including ELS, on epigenetic changes, should be studied.

**Table 1:** DNA methylation and early life stress.

Study	Population	Paradigm or model	Type of study	Gene(s) identifies	References
Animal	Mice	MS + SI	Bisulfite PCR, pyrosequencing	Nr3c1, Cacna1c	[114]
	Rats	Chronic mild stress	Methylation-specific real-time PCR	BDNF	[118]
	Mice	Administrated corticosterone	Bisulfite PCR, pyrosequencing	FKBP5	[119]
	Mice	MS + limited nesting material	ChIP-seq	H3K79me2	[120]
	Mice	Modified MS	MeDIP sequencing	DMRs in hypothalamus	[121]
	Mice	MS	Methylation-specific real-time PCR	p11	[122]
Clinical	Adolescents	Physical abuse, sexual abuse, physical neglect	Array-based genome-wide methylation	PSEN2, GRIN2D, SYNJ2	[123]
	Newborns	Maternal psychopathology, perceived stress, socioeconomic and psychosocial stress	MeDIP analysis, pyrosequencing	MORC1	[124]
	Newborns	Prenatal maternal mood	Bisulfite PCR, pyrosequencing	SLC6A4	[125]
	Newborns	Material deprivation, mundane stress, war stress	Bisulfite DNA sequencing	NR3C1	[126]
	Newborns	Prenatal maternal mood	Bisulfite DNA sequencing	NR3C1	[127]
	Newborns	Maternal stressful life events, maternal chronic stress experience, maternal depressive symptoms	Bisulfite DNA sequencing	OXTR	[128]
	Adolescents	Physical abuse, sexual abuse, emotional abuse, emotional neglect, physical neglect	Bisulfite PCR, high resolution melting analysis	OXT, OXTR	[129]
	Children	Child maltreatment	Array-based genome-wide methylation	DNA methylation age	[130]
	Adolescents	Physical abuse, sexual abuse, emotional abuse, emotional neglect, physical neglect	Array-based genome-wide methylation	DNA methylation age	[131]
	Newborns	Physical abuse, sexual abuse, emotional abuse, emotional neglect, physical neglect	Bisulfite PCR, pyrosequencing	FKBP5	[132]
	Adolescents	Interpersonal violence during grandmaternal pregnancy	Array-based genome-wide methylation	Five CpG sites	[133]
	Adolescents	Physical abuse, sexual abuse, neglect, emotional abuse, witnessed domestic violence	Array-based genome-wide methylation	ID3, GRIN1, TPPP	[134]
	Adults	Emotional abuse, emotional neglect, physical abuse, loneliness/psychological stress, parents’ discord, traumatic sexual experiences	Bisulfite PCR, pyrosequencing	FKBP5	[135]
	Adolescents	Physical abuse, sexual abuse, neglect, emotional abuse, witnessed domestic violence	Array-based genome-wide methylation	2868 CpG sites	[136]
	Adolescents	Physical abuse, emotional abuse, sexual abuse, witnessed physical violence, peer physical violence, physical neglect, emotional neglect	Array-based genome-wide methylation	hGR	[137]
	Adolescents	Neglect, physical abuse, sexual abuse, witnessed domestic violence	Array-based genome-wide methylation	OTX2	[138]
	Adolescents	Symptoms of depression and anxiety, psychosocial stress	Bisulfite PCR, pyrosequencing	NR3C1	[139]
	Adolescents	Physical abuse, sexual abuse, emotional abuse, domestic violence, other forms of interpersonal violence, emotional neglect, physical neglect, food insecurity, cognitive deprivation	Array-based genome-wide methylation	DNA methylation age	[140]
	Adolescents	Intimate partner violence	Genome-wide DNA methylation	NR3C1, FKBP5	[141]

*MS* maternal separation, *SI* social isolation, *MeDIP* methylation immunoprecipitation, *DMRs* differentially methylated regions.

**Table 2:** MicroRNA and early life stress.

Study	Population	Paradigm or model	Type of study	MicroRNAs	References
Animal	Rats	MS	qPCR	MiR-200a-3p, miR-320-5p, miR-322-5p	[184]
	Rats	MS	MicroRNA sequencing	MiR-29b-1-5p, miR-219a, miR-301b-3p,	[185]
	Mice	Chronic unpredictable mild stress	qPCR	MiR-15b	[186]
Clinical	Adults with BPD	Physical abuse, sexual abuse, emotional abuse, emotional neglect, physical neglect	Array-based genome-wide methylation	MiR-124	[187]
	Adults	Childhood trauma	MicroRNA microarray analysis	MiR-125b-1-3p	[188]
	Adults	Childhood abuse	MeDIP and microarray analysis	MiR-514, let-7d, miR-520c, miR-215, miR-519a, miR-519e, miR-203	[189]
	Adolescents	Physical abuse, sexual abuse, emotional abuse, emotional neglect, physical neglect	Genome-wide small RNA sequencing	MiR-450a-2-3p, miR-556-3p, miR-2115-3	[161]

*MS* maternal separation, *BPD* borderline personality disorder, *MeDIP* methylation immunoprecipitation.

Besides these limitations, several outstanding questions need answers: (1) Are there commonalities in ELS-associated methylation marks between adults and adolescents? (2) Do epigenetic changes affect mental and physical health in youth and beyond? (3) Are the changes sex-specific? (4) Are DNA methylation patterns heritable, and whether this can explain part of the heritability of depression? (5) Do different types of early life stresses have differential impacts on epigenetic programming, and are there shared vs. unique epigenetic signatures of maltreatment types? (6) Can these epigenetic signatures be used as predictors of different psychiatric illnesses? (7) Do prenatal and postnatal stresses commonly target epigenetic programming to impact mental health and behavior? (8) Can the epigenetic modifiers be used to reverse the clinical outcome? It is interesting to note that contrary to the finding in adults, Devlin et al. [125] reported no significant effect on BDNF methylation in adolescents with ELS, whereas Dukal et al. [190] reported that SLC6A4 methylation in females was significantly higher than in males, but it was not associated with ELS. On the other hand, Mckibben et al. [185] reported sex-specific changes in ELS-induced depression in several miRNAs. Using array-based genome-wide methylation, Wikenius et al. [191] found no significant associations between maternal depressive symptoms and DNA methylation in babies at 6 weeks and 12 months. Not related to ELS, Cardenas et al. [192] found that maternal use of antidepressants was associated with Zinc Finger Protein 575 gene (ZNF575) methylation in newborns but not in early childhood. Thus, the associations between ELS and epigenetic changes are quite complicated. Further studies will be needed to test the role of potential moderators in the identified associations, including the age of onset and chronicity of maltreatment exposure.

Animal studies have shown that the effects of caregiver deprivation on hypothalamic-pituitary-adrenal (HPA) axis function are dependent on the timing of exposure [193]. Animals separated on the third postnatal day demonstrate no immediate alterations in HPA function, whereas HPA responsiveness is markedly elevated in those separated on PND11. Additionally, examination of long-term alterations in stress reactivity indicated that animals separated on PND3 and PND11 showed hyper and hypo HPA responsiveness in adulthood [194]. These studies suggest that the timing of exposure to early-life trauma is critical in stress responsiveness. Whether such separation can be studied in humans is a matter of debate, given that most children exposed to early-life adversity continue to be exposed to adverse conditions throughout development.

In terms of treatment, Levine et al. [195] reported that histone deacetylases (HDACs) 1, 3, 7, 8, and 10 mRNA expressions were significantly decreased in the Balb/c mice ELS model and fluoxetine augmentated all histone modifications. Elmer et al. [196] reported that ketamine metabolite (2R,6R)-hydroxynorketamine reversed behavioral despair produced by adolescent trauma. Furthermore, recently, Wrobel et al. [197] examined the role of childhood trauma in treating bipolar disorder. The effect of childhood trauma on treatment outcomes was evaluated among participants randomized to treatment with lithium or quetiapine. It was found that although participants with a history of any childhood trauma presented with greater symptom severity and functional impairment at most study visits, participants with and without a history of any childhood trauma showed similar rates of improvement in symptom severity and functional impairment over the 24 weeks of treatment.

In conclusion, although several studies show that epigenetic modifications are critical in mediating the effect of ELS on various aspects of behavior in adulthood; however, a large body of work is needed to dissect the impact of ELS on mental disorders in adolescents. More recently, epigenetic contributions to the intergenerational and transgenerational heritability of mental disorders are actively being pursued [198–203]. This includes DNA modifications and non-coding RNAs, both contributing to brain circuitry changes and, ultimately, behavior. Depressive states in mothers have also been shown to be associated with their children’s functioning, including potential vulnerabilities to the onset of depression in their later lives [200]. ELS also mediates intergenerational and transgenerational transmission of epigenetic changes [198, 204], and can cause vulnerability to develop psychiatric diseases [205]. This area of research is of high significance and may shed light on the mechanisms of ELS-mediated development of depression and suicidal behavior in adolescents.

Epigenetics has emerged as an important potential target of treatment development. It remains to be seen if epigenetic changes will emerge as bona fide treatment targets. However, the development of molecular approaches that target specific epigenetic marks may ultimately treat specific environmentally-induced diseases. However, this approach may also improve a broad set of disorders that are affected by similar environmental antecedents. The epigenetic changes may serve as common and possibly modifiable vulnerability factors for trauma-related disorders more broadly. Targeting these changes might lead to novel treatments and, more importantly, ways of reversing the effects of ELS and thereby reducing the risk for a range of mental disorders. This could also bring a new generation of risk-modifying approaches that may provide ways of preventing and not just treating diseases.
